# Revealing taxonomy, activity, and substrate assimilation in mixed bacterial communities by GroEL-proteotyping-based stable isotope probing

**DOI:** 10.1016/j.isci.2024.111249

**Published:** 2024-10-28

**Authors:** Simon Klaes, Shobhit Madan, Darja Deobald, Myriel Cooper, Lorenz Adrian

**Affiliations:** 1 Department of Molecular Environmental Biotechnology, Helmholtz-Centre for Environmental Research – UFZ, 04318 Leipzig, Saxony, Germany; 2 Chair of Geobiotechnology, Technische Universität Berlin, 13355 Berlin, Berlin, Germany; 3 Faculty of Engineering, Ansbach University of Applied Sciences, 91522 Ansbach, Bavaria, Germany; 4 Chair of Environmental Microbiology, Technische Universität Berlin, 10587 Berlin, Berlin, Germany; 5 Agroecologie Department, Institut Agro Dijon, INRAE, University Bourgogne Franche-Comte, Bourgogne Franche-Comte, 21000 Dijon, France

**Keywords:** Biological sciences, Microbiology, Proteomics, Microbiological method

## Abstract

Protein-based stable isotope probing (protein-SIP) can link microbial taxa to substrate assimilation. Traditionally, protein-SIP requires a sample-specific metagenome-derived database for samples with unknown composition. Here, we describe GroEL-prototyping-based stable isotope probing (GroEL-SIP), that uses GroEL as a taxonomic marker protein to identify bacterial taxa (GroEL-proteotyping) coupled to SIP directly linking identified taxa to substrate consumption. GroEL-SIP’s main advantages are that (1) it can be performed with a sample-independent database and (2) sample complexity can be reduced by enriching GroEL proteins, increasing sensitivity and reducing instrument time. We applied GroEL-SIP to pure cultures, synthetic bicultures, and a human gut model using ^2^H-, ^18^O-, and ^13^C-labeled substrates. While ^2^H and ^18^O allowed assessing general activity, ^13^C enabled differentiation of substrate source and utilized metabolic pathways. GroEL-SIP offers fast and straightforward protein-SIP analyses of highly abundant families in mixed bacterial communities, but further work is needed to improve sensitivity, resolution, and database coverage.

## Highlights

• GroEL-proteotyping-based stable isotope probing (GroEL-SIP) links activity to bacterial taxa
• GroEL-SIP uses a universal, sample-independent database
• GroEL-SIP allows cost-efficient and fast SIP analyses of abundant bacterial families
• GroEL serves as a proxy for isotope incorporation into the whole proteome

## Graphical abstract

**Figure:**
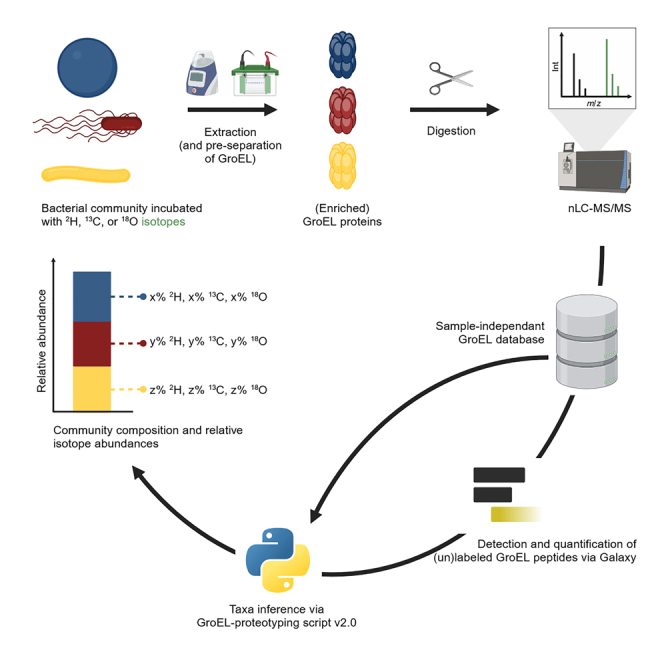

## Introduction

Microorganisms are integral to biotechnological applications,^1^ human health,^2^ and global biogeochemical cycles.^3^ In nature, microorganisms mostly live in complex microbial communities that govern their functionality.^4^ To understand the role of specific microbial groups within a community, different strategies are used. These include traditional isolation and cultivation approaches,^5^^,^^6^^,^^7^ taxonomic descriptions of microbial community changes over time^8^^,^^9^^,^^10^ and, more recently, stable isotope probing (SIP) techniques to link taxa to key functions in mixed microbial communities.^11^

In SIP experiments, microbial communities or single organisms are incubated with a substrate labeled with stable isotopes of the elements ^2^H,^12^^,^^13^^,^^14^^,^^15^ ^13^C,^16^^,^^17^^,^^18^^,^^19^^,^^20^^,^^21^ ^15^N,^13^^,^^17^ ^18^O^15^ or ^34^S.^22^ When the labeled substrate is metabolized, the stable isotope can get incorporated into different classes of biomolecules, such as DNA,^18^^,^^19^ RNA,^11^^,^^18^ fatty acids,^20^^,^^21^ or proteins.^16^^,^^17^ Subsequently, the mass of those biomolecules can be analyzed, and stable isotopes link the labeled taxa directly to a general (for ^2^H and ^18^O) or substrate-specific (for ^13^C, ^15^N, or ^34^S) activity. By this, SIP can identify key degraders,^23^^,^^24^ unravel metabolic pathways,^22^^,^^25^^,^^26^ and elucidate trophic relationships within microbial communities.^18^^,^^27^

The taxonomic resolution and the sensitivity of isotope detection differ depending on the investigated biomolecule (reviewed in^28^). For DNA/RNA-based methods, a high percentage of isotope incorporation is required to reliably separate labeled and non-labeled molecules. Furthermore, DNA/RNA are considered to be less abundant cell components than proteins and lipids.^29^^,^^30^ While diacyl phospholipids are abundant in bacteria, the incorporated phospolipid-derived fatty acids (PLFA) used for mass spectrometric isotope quantification show limited taxonomic information. Protein-SIP has the major advantage of sensitively detecting and quantifying isotope incorporation while preserving the taxonomic information. Furthermore, it has been suggested that estimates of biomass contributions are more accurate based on proteins than based on gene copy numbers.^31^

In protein-SIP, proteins are identified based on database matches. For pure cultures, databases can be derived directly from the organism’s genome. For unknown microbial communities, protein-SIP traditionally has to be accompanied by metagenome sequencing, which is demanding in cost, time, and bioinformatics skills and must be repeated for each new sample site. Recently, it has been shown that protein-based taxonomic profiles of unknown bacterial communities can also be obtained using whole broad-spectrum databases such as NCBInr or UniProtKB/Swiss-Prot (“proteotyping”)^32^^,^^33^^,^^34^^,^^35^^,^^36^^,^^37^ or subsets of those databases focusing on taxonomic marker proteins such as GroEL or elongation factors (“targeted proteotyping”).^38^^,^^39^^,^^40^ Whole broad-spectrum databases have comparable taxonomic resolution to metagenome-derived databases, but lead to high computation times due to their size.^31^^,^^33^ Using taxonomic marker protein databases can reduce computation times at the cost of lower taxonomic resolution. Taxonomic marker proteins are proteins that are abundant in all taxa of interest and contain phylogenetic information in their sequence to allow inferring the taxonomy of organisms in a targeted proteotyping approach. The taxonomic resolution can depend on the protein and the taxa of interest. Elongation factors have been used for proteotyping domain level abundances across soil taxa.^38^ Furthermore, elongation factors were combined with ribosomal proteins to assess human gut microbiota at class level.^39^ The highest taxonomic resolution was reported using GroEL for proteotyping bacteria at family level in mock communities and human gut samples.^40^

Protein-SIP of complex communities has been performed successfully with the large NCBInr database.^16^^,^^41^^,^^42^^,^^43^^,^^44^ Furthermore, when using a metagenome-derived database for protein-SIP, the taxonomic profiles based on all identified labeled proteins and a subset of identified taxonomic marker proteins containing GroEL were similar.^45^ However, it remains unclear whether protein-SIP can effectively be performed with a taxonomic marker protein database, omitting the need for metagenome sequencing while preserving acceptable taxonomic resolution and computation time.

Here, we explore whether GroEL-proteotyping can be combined with SIP to infer active microbial taxa in unknown samples directly from mass spectrometric data using a sample-independent database. For this purpose, we (1) modified a previously published GroEL-proteotyping Python script to render stable isotope incorporation rates in GroEL peptides of identified taxa, (2) tested GroEL-based stable isotope probing (GroEL-SIP) to identify actively degrading organisms in pure cultures, synthetic bicultures, and an *in vitro* model of the human distal gut microbial ecosystem using ^2^H-, ^18^O-, and ^13^C-labeled substrate, and (3) distinguished the transformation pathways of labeled benzoate catalyzed by *Thauera aromatica* K172 and *Pseudomonas putida* KT2440 in model bicultures.

## Results

### Incorporation of ^13^C into GroEL of *T. aromatica* K172

To explore the suitability of GroEL peptides as a proxy for isotope incorporation from a substrate into the whole proteome, we mass-analyzed synthetic mixtures of labeled and unlabeled *T. aromatica* K172 proteins. The resulting proteomic data was evaluated with two databases: (1) the whole proteome database of *T. aromatica* K172 and (2) our GroEL database (Table 1).

**Table 1:** Number of identified peptides, as well as relative isotope abundance (RIA) and labeling ratio (LR) of identified labeled peptides from protein extracts of *Thauera aromatica* K172, cultivated with 5 mM α-^13^C-benzoate or ^12^C-benzoate mixed in different ratios

Relative abundance of ^13^C-labeled proteins in the protein mixture [%]	Search against the whole proteome database (Requires prior knowledge of the sample’s community composition)				Search against the GroEL database and filtering with a Top Rank Count threshold of ≥ 5 (Does not require prior knowledge of the sample’s community composition)			
	Total identified peptides	Identified labeled peptides	Median RIA of labeled peptides [%]	Median LR of labeled peptides [%]	Total identified peptides	Identified labeled peptides	Median RIA of labeled peptides [%]	Median LR of labeled peptides [%]
0	2476.7 ± 254.5	3.7 ± 0.6	3.4 ± 1.2	50.0 ± 0.0	14.7 ± 0.5	0 ± 0	0 ± 0	0 ± 0
25	1746.3 ± 181.9	326.0 ± 32.8	12.6 ± 0.1	24.1 ± 1.3	15.7 ± 1.5	12.0 ± 2.0	14.1 ± 0.8	24.3 ± 2.1
50	1549.1 ± 356.5	743.7 ± 171.5	12.2 ± 0.0	50.9 ± 3.0	15.2 ± 1.2	13.7 ± 0.5	12.5 ± 0.8	52.3 ± 3.3
75	484.3 ± 287.1	402.3 ± 226.0	12.4 ± 0.3	73.9 ± 2.5	10.0 ± 2.6	10.0 ± 2.6	11.7 ± 0.4	74.6 ± 2.7
100	0 ± 0	0 ± 0	0 ± 0	0 ± 0	0 ± 0	0 ± 0	0 ± 0	0 ± 0

Peptides were identified using the whole proteome database of *Thauera aromatica* K172 containing 3,335 entries or the GroEL database with 72,759 non-redundant entries. Peptides were counted as labeled when at least one RIA was detected in addition to the natural RIA. If MetaProSIP reported multiple RIAs for a peptide, only the highest RIA was used to calculate the median and LR. Values represent means of biological triplicates ± standard deviations.

When using the whole proteome database, the total number of identified peptides decreased with an increasing amount of ^13^C-labeled proteins in the mixture. In the case of 100% ^13^C-labeled proteins, no peptides were identified with either of the two databases because the identification via the MetaProSIP algorithm requires the presence of unlabeled peptides. The highest number of labeled peptides (743.7 ± 171.5) was identified at 50% relative abundance of ^13^C-labeled proteins. Therefore, a mixing ratio of 50% was used in the following experiments. The labeling ratio reflected the input of ^13^C-labeled proteins except for purely labeled (100%) and purely unlabeled (0%) samples due to no or false-positive identifications, respectively. The detected RIA values were slightly lower than the theoretical α-^13^C-benzoate RIA value of 14.3%, indicating heterotrophic CO_2_ fixation.

We then compared the GroEL-based analysis with the whole proteome-based analysis. We detected ^13^C-labeled GroEL peptides with both databases, showing that ^13^C is incorporated into GroEL and can be identified in GroEL alone. Furthermore, the RIA and LR of labeled GroEL peptides detected with the GroEL database were similar to those determined for all labeled peptides with the whole proteome database, indicating that GroEL is a suitable proxy for stable isotope incorporation into the proteome.

### Differentiation of a co-cultivated biculture based on the used carbon source by tracing the incorporation of ^13^C-labeled substrate into GroEL

To evaluate the efficacy of GroEL-SIP in differentiating the participation of different bacteria in a biochemical transformation, we conducted a time-dependent analysis of a biculture consisting of *Escherichia coli* BL21(DE3) and *T. aromatica* K172. *T. aromatica* K172 is described to grow on benzoate as the sole carbon source.^46^ In contrast*, E. coli BL21*(DE3) has not been described to grow with benzoate as the carbon source. Indeed, when grown in the presence of ^12^C-acetate and α-^13^C-benzoate *E. coli* BL21(DE3) did not incorporate ^13^C into its proteome (Table S1).

To establish a biculture, we inoculated medium containing 5 mM α-^13^C-benzoate and 5 mM non-labeled acetate with *E. coli* BL21(DE3). After reaching the stationary phase, we injected *T. aromatica* K172 and continued incubation. Cells were collected from the biculture at designated time points. GroEL proteins were enriched relative to other proteins by SDS-PAGE before tryptic digestion and mass spectrometry. The identified peptides were associated with GroEL protein groups and filtered using a Top Rank Count threshold of ≥ 5. Taxonomy was inferred at genus level. The genera *Escherichia* and *Shigella* could not be differentiated with our approach and will be referred to as *Escherichia* in the following.

**Figure 1:**
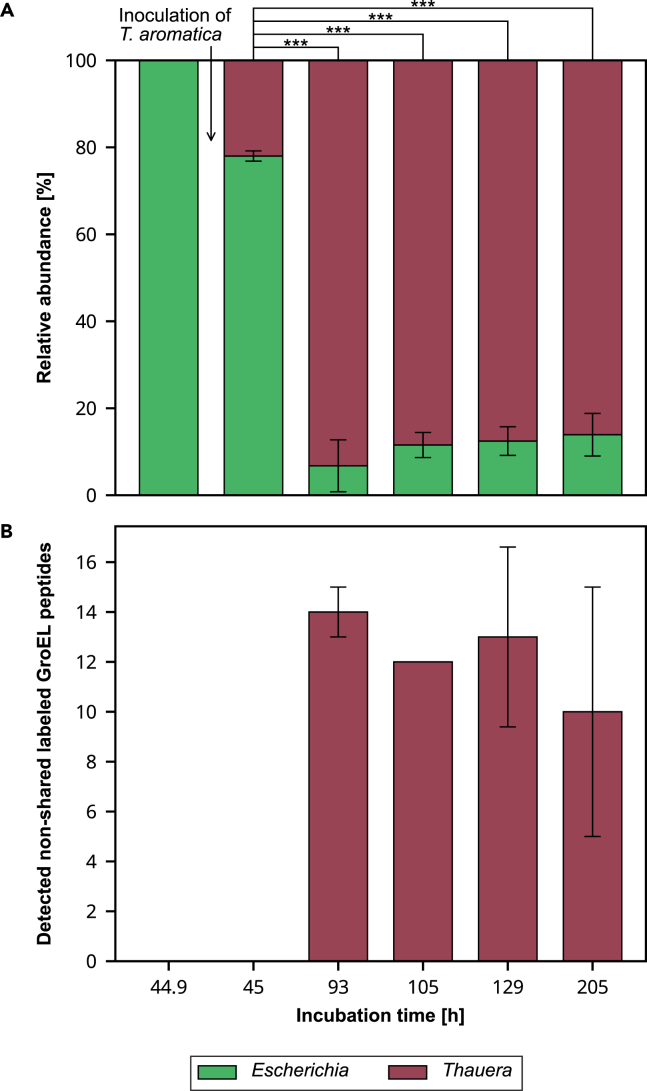
GroEL-based stable isotope probing of a co-cultivated biculture consisting of *Escherichia coli* BL21(DE3) and *Thauera aromatica* K172 at different time points Cultivation medium DSMZ 586 containing 5 mM α-^13^C-benzoate and 5 mM acetate was inoculated with *E. coli* BL21(DE3) to initiate the experiment, followed by the injection of *T. aromatica* K172 after 44.9 h of incubation. Bars represent means of biological triplicates. Error bars denote standard deviations. (A) Relative taxa abundances based on the sum of the precursor intensities of detected GroEL-peptides. Statistically significant differences in relative abundance between timepoints are indicated with ‘∗∗∗’ based on Student’s *t* test for the means of two independent samples with *p* < 0.001. (B) Number of detected labeled GroEL peptides not shared between both genera.

*Escherichia* was consistently detected at all investigated time points being the major contributor to the biculture during the initial 45 h of incubation (Figure 1A). *Thauera* was not detected until its injection at 45 h of incubation. After 93 h of total incubation time, the composition changed significantly (*p* < 0.001) with *Thauera* becoming the major contributor to the biculture reaching a relative abundance of up to 93.2 ± 6.0%. The significant change in relative abundance of *Thauera* after 93 h incubation indicates its growth and the success of co-cultivation. The majority of detected labeled peptides was non-shared between both genera at all investigated time points with a maximum of four shared labeled peptides (Table S2). In the first 45 h, no isotopically labeled peptides were detected (Figure 1B). After 93 h, isotopically labeled peptides that were not shared between *Thauera* and *Escherichia* were detected for *Thauera* but not for *Escherichia*. This demonstrates *T. aromatica* assimilated the labeled benzoate, whereas *E. coli* used only unlabeled acetate as a carbon source for growth. The median RIA of labeled GroEL peptides assigned to *Thauera* was very consistent and ranged from 12.0 ± 1.6% to 12.5 ± 1.7%, with a median LR between 40.3 ± 1.3% and 57.4 ± 2.4%. During bicultivation, RIA (*p* > 0.624) and LR (*p* > 0.945) values of labeled GroEL peptides did not differ significantly from pure cultures (Table 1), indicating that *T. aromatica* did not use acetate as a carbon source during the co-cultivation.

### Distinguishing metabolic pathways in a co-cultivated biculture by the detection of specific ^13^C-atom incorporation patterns in GroEL peptides

To assess the applicability of GroEL-SIP to distinguish organisms based on their catalyzed metabolic pathways, we performed a time course analysis of a co-cultured biculture comprising *T. aromatica* K172 and *P. putida* KT2440. The two strains were selected based on different benzoate degradation pathways: whereas *T. aromatica* degrades benzoate via the benzoyl-CoA pathway,^47^ *P. putida* degrades benzoate via the β-ketoadipate pathway,^48^ which leads to the removal of the ^13^C from the labeled α-^13^C-benzoate before its integration into central pathways. In pure cultures, both organisms grew on α-^13^C-benzoate as the sole carbon source. In agreement with the degradation pathways, isotopically labeled peptides were detected only for *T. aromatica* (Table S1).

We tested whether the degradation pathways can be distinguished in a co-cultivated biculture of the two strains based on isotope integration patterns. For this purpose, cells from the biculture were harvested at specified intervals, and crude protein extracts were prepared before enriching GroEL proteins by SDS-PAGE and mass spectrometry of tryptic digests. Mass spectra were matched with our GroEL database, and analyzed with our Python script at genus level.

**Figure 2:**
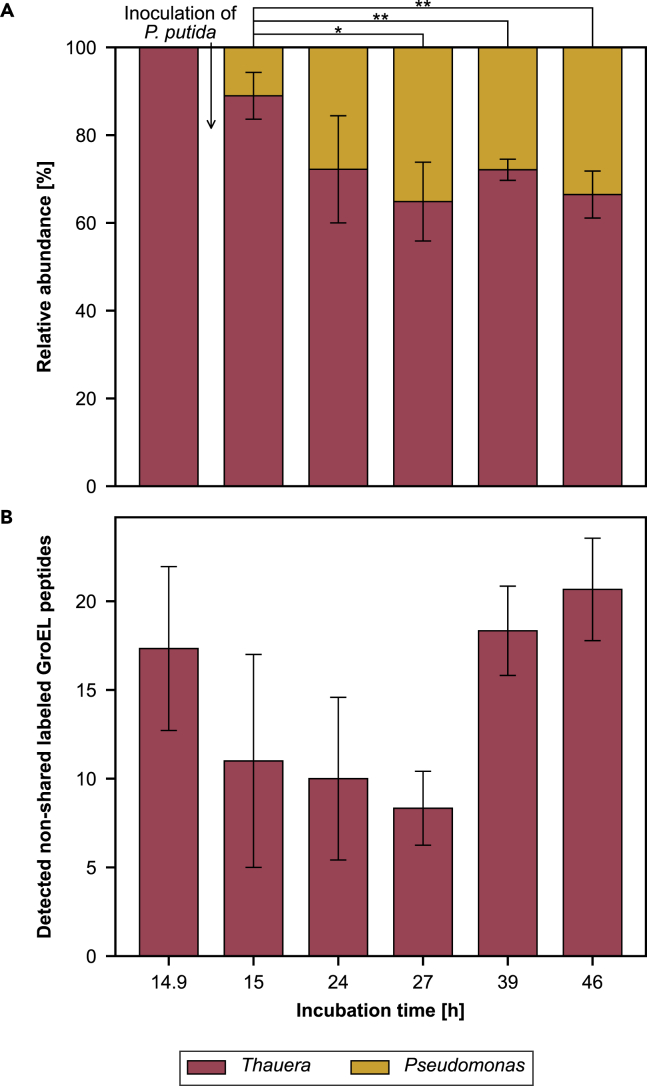
GroEL-based stable isotope probing of a co-cultivated biculture consisting of *Thauera aromatica* K172 and *Pseudomonas putida* KT2440 at different time points The cultivation medium, DSMZ 586, containing 5 mM α-^13^C-benzoate, was initially inoculated with *T. aromatica* K172, followed by the injection of *P. putida* KT2440 after 14.9 h of incubation. Bars represent means of biological triplicates. Error bars denote standard deviations. (A) Relative taxa abundances were determined based on the sum of the precursor intensities of detected GroEL-peptides. Statistically significant differences in relative abundance between timepoints are indicated with ‘∗’, ‘∗∗’ based on Student’s *t* test for the means of two independent samples with *p* < 0.05, and *p* < 0.01, respectively. (B) The number of detected labeled GroEL peptides not shared between both genera was assessed.

Our data show that the two strains can be distinguished in a co-culture based on the different degradation pathways using GroEL-SIP. *Thauera* was detected at all investigated time points as the most abundant genus (Figure 2A). *Pseudomonas* was detected after the injection of *P. putida* at 15 h total incubation time with a relative abundance of approximately 11.0 ± 5.3%. After 27 h of total incubation, the relative abundance of *Pseudomonas* increased significantly (*p* < 0.05) to up to 35.1 ± 9.0% indicating its growth and successful co-cultivation.

Throughout all examined time points, a maximum of three isotopically labeled peptides shared between both genera was detected (Table S3). Non-shared isotopically labeled peptides were exclusively identified for *Thauera* with a median RIA of 12.0 ± 0.9% to 13.8 ± 0.7% and median LR of 37.1 ± 18.6% to 69.4 ± 7.5%, suggesting that *T. aromatica* incorporated the α-C-atom of benzoate (Figure 2B). During bicultivation, RIA (*p* > 0.639) and LR (*p* > 0.667) values of labeled GroEL peptides did not differ significantly from pure culture. While GroEL peptides from *Pseudomonas* were detected, none of them exhibited non-shared labeling, suggesting growth without the incorporation of the α-C-atom of benzoate. The detection of stable isotopes in GroEL peptides of *Thauera* but not in those of *Pseudomonas* aligns with our observations in pure culture experiments (Table S1).

In conclusion, our data demonstrate that GroEL-SIP can trace the composition of bacterial bicultures, simultaneously differentiating taxa by their metabolic pathways using substrate isotopically labeled at positions of interest.

### Case study: Investigating family-specific metabolic activity in complex communities through tracing ^2^H and ^18^O incorporation into GroEL

In the preceding experiments, we restricted ourselves to defined communities consisting of only two bacterial species. To assess the applicability of GroEL-SIP in characterizing complex bacterial communities and to test our approach with other isotopes, such as ^2^H and ^18^O, we analyzed a previously published metaproteomic dataset.^15^^,^^49^ The dataset was initially generated by Starke et al. to investigate the response of gut microorganisms in metabolic activity to a fiber-rich and protein-rich diet. Specifically, the dataset was derived from an *in vitro* model community mimicking the ecosystem of the human distal gut. The community, consisting of 63 species from 22 families, was cultivated with either ^2^H_2_O or H_2_^18^O to assess taxon-specific metabolic activity. Additionally, the community was incubated with fiber-rich or protein-rich medium to simulate different dietary preferences. For each isotopic variant and each dietary condition, triplicates were incubated for 12 h in a mixture of 75% unlabeled and 25% labeled water. In the controls, the water was exclusively unlabeled. The original study used a metagenome-derived database and MetaProSIP to identify the 20 most abundant species belonging to 10 different families. The original study’s results indicated that ^18^O was stronger incorporated than ^2^H. The communities were dominated by species of the families *Bacteroidaceae* and *Akkermansiaceae,* with most labeled peptides assigned to these two families, irrespective of the medium composition.^15^ The goal of our analysis was to evaluate how similar results would be if the published mass spectrometric data was analyzed exclusively with our pre-calculated GroEL database instead of the comprehensive metagenome database.

**Figure 3:**
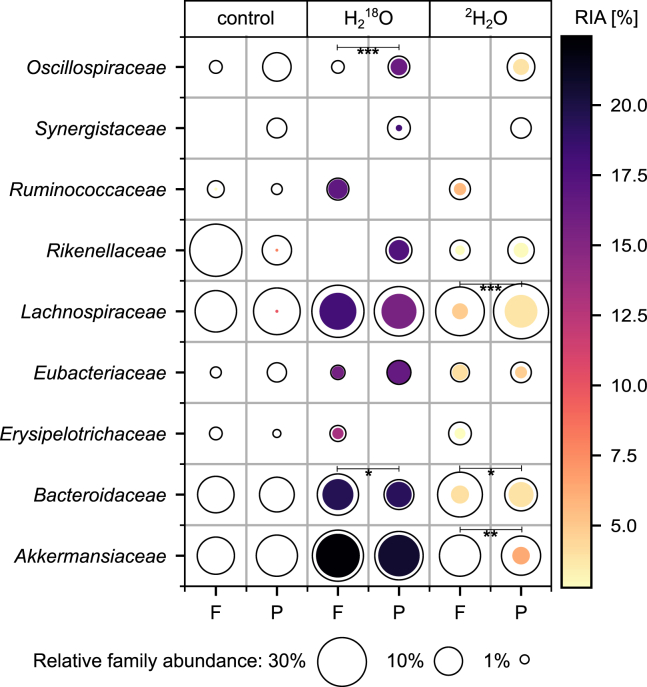
Characterizing the taxonomic composition and stable isotope incorporation in an *in vitro* model of the human gut under a fiber-rich (F) or protein-rich (P) diet using GroEL-SIP The metaproteome dataset, originally obtained by Starke et al.^15^ and published by Kleiner et al.,^49^ served as the basis for this analysis. The unfilled circles’ areas (black outlines) represent relative family abundances based on the sum of the precursor intensities of identified GroEL peptides. The colored area within each circle indicates the abundance of the isotopically labeled peptides within the respective family peptides: it shows the product of the relative count of labeled GroEL peptides and the relative family abundance. The tones of the colored areas reflect the median relative isotope abundance (RIA) of the detected labeled GroEL peptides. Families were included in the plot only if they were detected in at least two of three biological replicates. For families detected under both diets, statistically significant differences in the relative count of labeled GroEL peptides are indicated with ‘∗’, ‘∗∗’, and ‘∗∗∗’ based on Student’s *t* test on the means of replicates at *p* < 0.05; *p* < 0.01; and *p* < 0.001, respectively.

In our analysis, we assigned identified labeled and unlabeled peptides to GroEL protein groups, refined these groups using a Top Rank Count filter with a threshold of ≥5 and assessed the taxonomy at the family level to ensure a high level of specificity. After filtering, we identified nine families in at least two of three biological replicates, all of which were also identified in the original study (Figure 3). Notably, eight of these families belonged to the most abundant families. However, we did not reliably detect two of the ten most abundant families, i.e., *Clostridiaceae* and *Acidaminococcaceae*. Whereas *Acidaminococcaceae* was not detected in any sample, *Clostridiaceae* was only detected in single samples from the biological triplicates. Diverging from the original study, we detected certain families exclusively in one of the two dietary conditions. For instance, *Synergistaceae* was only identified in the protein-rich medium. Furthermore, some families were reliably detected only in the unlabeled controls for both dietary conditions.

In general, we found higher RIA values of GroEL peptides for the incorporation of ^18^O compared to ^2^H. For families detected labeled under both dietary conditions, RIA values differed significantly (*p* < 0.05) between the diets only in the ^2^H_2_O setup and only for two families: While GroEL peptides assigned to *Lachnospiraceae* had higher RIA values in the fiber-rich medium (4.9 ± 0.0% vs. 3.8 ± 0.5%) GroEL peptides assigned to *Eubacteriaceae* had higher RIA values in the protein-rich medium (4.7 ± 0.3% vs. 4.1 ± 0.2%). The relative count of labeled GroEL peptides differed significantly between the dietary conditions for some families, indicating changes in activity. In the ^2^H_2_O setup, *Bacteroidaceae*, *Lachnospiraceae*, and *Akkermansiaceae* exhibited substantially higher activity in the protein-rich medium than in the fiber-rich medium. Furthermore, for *Oscillospiraceae* labeled GroEL peptides were only detected in the protein-rich medium. GroEL peptides for *Ruminococcaceae* and *Erysipelotrichaceae* were only detected in fiber-rich setups when heavy water was used. These GroEL peptides were highly labeled, indicating enhanced activity of *Ruminococcaceae* and *Erysipelotrichaceae* in the fiber-rich medium.

In conclusion, we show that GroEL-SIP can identify dominant families and quantify stable isotope incorporation for both ^2^H and ^18^O in complex communities. However, we also see limitations of our method. Identification of highly abundant families was incomplete and the detection of low abundant families is challenging, particularly in the presence of labeled peptides.

## Discussion

Our findings show that GroEL can be used as a proxy for measuring isotope incorporation into the proteome of replicating organisms. Thus, GroEL-proteotyping can be combined with SIP, allowing protein-SIP experiments with a taxonomic marker protein database. We refer to our approach as “GroEL-proteotyping-based stable isotope probing” (GroEL-SIP). GroEL-SIP has the major advantage that it uses a sample-independent database with 72,759 entries that is four magnitudes smaller than the NCBInr database with 631,584,287 entries (accessed 2023/11/09), reducing computation time while still omitting efforts and costs for creating a metagenome-derived database. This can be especially valuable when repeated timely monitoring of systems is desired, for example, during reactor cultivation.

Furthermore, GroEL proteins can be selectively enriched from cell crude extracts by SDS-PAGE, verifying successful protein extraction, reducing sample complexity and leading to a 1.8-fold increase in the number of detected GroEL peptides.^40^ Alternatively, GroEL proteins could be enriched by size exclusion chromatography or molecular weight cutoff filters. However, GroEL-SIP has a lower taxonomic resolution than approaches focusing on all proteins: whereas a metagenome-derived or the NCBInr database allows characterizations down to the species level, GroEL-SIP only achieved family level resolution in complex communities and genus-level resolution in defined bicultures.^31^^,^^33^^,^^40^ Compared to SIP approaches based on other biomolecules, GroEL-SIP provides higher taxonomic resolution than PLFA-SIP, while still allowing precise quantification of isotope incorporation that is not possible by DNA/RNA-SIP, although DNA/RNA-SIP offer the highest taxonomic resolution.^28^ While the taxonomic resolution of GroEL-SIP can be sufficient for some use cases, such as highly enriched cultures with one target organism or continuous bioreactors, subsequent studies should try to enhance taxonomic resolution, e.g., by combining multiple taxonomic marker proteins. The resolution and sensitivity could also be improved by using Sipros 4, which has recently been reported to outperform MetaProSIP in detecting ^13^C- and ^15^N-labeled peptides at comparable speed.^50^

In our biculture experiments, GroEL-SIP successfully characterized the taxonomic composition of the bicultures while simultaneously tracing the carbon flow from benzoate’s α-C-atom, which allowed monitoring substrate-specific activity and differentiation of catalyzed metabolic pathways. Our results confirmed that *T. aromatica* K172^46^ but not *E. coli* BL21(DE3)^51^ grows on benzoate as the carbon source. Furthermore, our analysis validated that benzoate is aerobically degraded by *T. aromatica* via the benzoyl-CoA pathway^47^ leading to an incorporation of the α-C-atom and by *P. putida* using the β-ketoadipate pathway via catechol^48^ resulting in the cleavage of the α-C-atom. Whereas a substrate labeled at a specific position of interest can be especially useful for elucidating metabolic pathways, a fully labeled substrate should be preferred when analyzing substrate consumption capacities. The investigation of the human distal gut model shows that GroEL-SIP based on heavy water isotopes can assess the general activity of dominant families in mixed bacterial communities. GroEL-SIP identified more families in the unlabeled controls than in heavy water treatments. Some families were detected at low abundance in unlabeled controls but not detected in heavy water treatments, indicating that the presence of isotopically labeled peptides decreases the sensitivity of GroEL-proteotyping. The original study from Starke et al. used MetaProSIP and a metagenome-derived database but did not find significant differences in taxon-specific activity related to diet change.^15^ The same dataset was reanalyzed by Kleiner et al. using Calis-p.^49^ Kleiner et al. found an enhanced activity of species from the families *Bacteroidaceae*, *Lachnospiraceae*, *Akkermansiaceae*, and *Enterobacteriaceae* in protein-rich compared to fiber-rich conditions. We also detected increased activity of the families *Bacteroidaceae*, *Lachnospiraceae*, and *Akkermansiaceae, but Oscillospiraceae instead of Enterobacteriaceae.* We did not identify *Enterobacteriaceae* at all. Similarly, *Enterobacteriaceae* were not within the 20 most abundant taxa of the community described by Starke et al..^15^ In summary, GroEL-SIP detected similar family abundances and isotope incorporation as the two previous analyses, but there were also significant differences between all the three computational analyses. More work is needed to differentiate the strengths and weaknesses of the various approaches and there is no clear conclusion which approach describes the reality best with respect to different parameters (population shares, isotope incorporation, changes though medium type). We are currently not recommending GroEL-SIP as an application-ready approach for the analysis of complex communities, but we have obtained encouraging results to further explore and refine the method.

Cross-feeding is a phenomenon in which primary consumers or dead cells release isotopically labeled intermediates that are taken up by other microorganisms, leading to their isotopic labeling. Cross-feeding can provide insights into food webs,^18^^,^^27^ but it also has the potential to misidentify primary consumers.^52^ Although we did not observe any cross-feeding during our biculture experiments, we cannot rule out its occurrence in the human distal gut model. Cross-feeding phenomena can be more accurately resolved by analyzing the shape of the isotopic distribution and conducting detailed time-resolved analyses, which require subsequent measurements, high costs, and extensive instrument time.^53^ Given that GroEL-SIP can reduce costs and instrument time compared to conventional SIP analyses, it could be particularly advantageous for studying cross-feeding phenomena. However, for those seeking precise quantification of taxa contributions, we recommend using metagenome-derived databases.^31^

In GroEL-SIP, detecting labeled organisms relies on the incorporation of isotopes into GroEL, which requires both metabolization of the labeled substrate and GroEL protein synthesis. Since GroEL is essential for most bacteria^54^ (except for certain *Mycoplasma* and *Ureaplasma* strains^55^), its synthesis is typically indirectly required for bacterial replication. Therefore, GroEL-SIP can use GroEL as a proxy for isotope incorporation in replicating bacteria. However, if the labeled substrate is metabolized in non-replicating bacteria to synthesize proteins other than GroEL, GroEL-SIP cannot detect these partially labeled organisms. In such cases, SIP based on the whole proteome would be an appropriate approach.

In principle, GroEL-SIP allows SIP analyses of active and highly abundant bacterial taxa with a pre-computed and extendable database. We expect that the number of available GroEL sequences in public databases will further increase in the next years, improving the size and coverage of our GroEL database. Currently, GroEL-SIP has a lower computation time but also a lower sensitivity and taxonomic resolution than conventional protein-SIP analyses with metagenome-derived or NCBInr databases. Furthermore, analyzing environmental samples remains challenging because the current protein/taxa inference of GroEL-SIP relies on perfect peptide matches with the database. Non-sequenced organisms can be present in environmental samples, and differences in amino acid sequences compared to the database are likely. With our current strategy, these constraints will reduce the calculated Top Rank Count and, thus, the sensitivity for detecting taxa in environmental samples. Nevertheless, we are convinced that adapted protein and taxa inference strategies, as well as the increasing growth of public protein data and enhanced data acquisition by mass spectrometers can overcome these challenges and prospectively make GroEL-SIP a valuable alternative to traditional SIP approaches, especially when a timely and cost-efficient monitoring of highly abundant taxa in different samples is necessary. In its current development stage, GroEL-SIP is well suited for isotope incorporation studies in defined cultures.

### Limitations of the study

This study introduces the concept of GroEL-SIP on defined bacterial bicultures (using ^13^C) and more complex bacterial communities (using ^2^H and ^18^O). However, further validation is needed across additional datasets and sample matrices. Moreover, the low taxonomic resolution of GroEL-proteotyping remains unresolved and should be addressed in future research.

## Resource availability

### Lead contact

Further information and requests for resources and reagents should be directed to and will be fulfilled by S.K. (simon.klaes@ufz.de).

### Materials availability

This study did not generate new unique reagents.

### Data and code availability

• Raw and processed mass spectrometry proteomic data generated in this study have been deposited to the ProteomeXchange Consortium (http://proteomecentral.proteomexchange.org) via the PRIDE partner repository^56^ with the dataset identifier PRIDE: PXD048255 and are publicly available as of date of publication. In addition, this paper analyzes existing, publicly available data, accessible via the PRIDE partner repository as PRIDE: PXD024291.
• All original code has been deposited to Zenodo at https://doi.org/10.5281/zenodo.13965049 and is publicly available as of the date of publication.
• Any additional information required to reanalyze the data reported in this paper is available from the lead contact upon request

## Acknowledgments

Proteomics raw data published here was acquired at UFZ at the Center for Chemical Microscopy (ProVIS), which is supported by the 10.13039/501100008530European Regional Development Fund (EFRE) and the Hemholtz Association. We acknowledge Benjamin Scheer for his assistance with mass spectrometric measurements. We thank Matthias Bernt for support on the Galaxy server and the contributors of MS-GF + for their rapid troubleshooting. This research was funded by the 10.13039/501100001659German Research Foundation (DFG) grant number GRK 2032/2. Open Access funding enabled and organized by Projekt DEAL. The graphical abstract was created with BioRender.com.

## Author contributions

Conceptualization, S.K., D.D., M.C., and L.A.; Data curation, S.K.; Formal analysis, S.K.; Funding acquisition, M.C. and L.A.; Investigation, S.K. and S.M.; Methodology, S.K. and D.D.; Software, S.K. and L.A.; Validation, S.K. and S.M.; Visualization, S.K.; Writing – original draft, S.K.; Writing – review and editing, S.K., S.M., D.D., M.C., and L.A.

## Declaration of interests

The authors declare no competing interests.

## STAR★Methods

### Key resources table

**Table:** 

REAGENT or RESOURCE	SOURCE	IDENTIFIER
**Bacterial and Virus Strains**		

*Pseudomonas putida* KT2440	German collection of microorganisms and cell cultures (DSMZ)	DSM 6125
*Thauera aromatica* K172	German collection of microorganisms and cell cultures (DSMZ)	DSM 6984
*Escherichia coli* BL21(DE3)	Thermo Scientific	Cat#EC0114

**Chemicals, Peptides, and Recombinant Proteins**		

α-^13^C-benzoic acid	Eurisotop	CAS 3880-99-7
2-Iodoacetamide	Merck	CAS 144-48-9
Ammonium bicarbonate	Bernd Kraft	CAS 1066-33-7
Bovine serum albumin	Thermo Scientific	Cat#23209
Dithiothreitol	ITW Reagents	CAS 3483-12-3
Sequencing-grade modified trypsin	Promega	Cat#V5111
Sodium acetate	Sigma-Aldrich	CAS 127-09-3
Sodium benzoate	Sigma-Aldrich	CAS 532-32-1

**Critical Commercial Assays**		

Pierce BCA protein assay kits	Thermo Scientific	Cat#23225

**Deposited Data**		

Metaproteome data from an *in vitro* model of the human distal gut^15^^,^^49^	PRIDE repository	PRIDE: PXD024291
Metaproteome data generated in this study	This paper	PRIDE: PXD048255

**Software and Algorithms**		

Galaxy (UFZ instance)	The Galaxy Community, 2024^57^	https://galaxy.intranet.ufz.de/
GroEL-proteotyping script v2.0	This paper	https://doi.org/10.5281/zenodo.13965049
MS-GF + v2023.01.12	Kim and Pevzner, 2014^58^	https://github.com/MSGFPlus/msgfplus
ProteoWizard v3.0.19046	Kessner et al., 2008^59^	https://proteowizard.sourceforge.io/
OpenMS 2.8	Röst et al., 2016^60^	https://openms.de/

### Experimental model and study participant details

*Pseudomonas putida* KT2440 and *Thauera aromatica* K172 were provided by the German collection of microorganisms and cell cultures, DSMZ. *Escherichia coli* BL21(DE3) was obtained from Thermo Scientific. All organisms were cultivated in 100 mL cotton-plugged Erlenmeyer flasks with 50 mL of nitrate-free DSMZ 586 medium with 5 mM sodium α-^13^C-benzoate at 30°C under oxic conditions on a rotary shaker in the dark. For unlabeled controls, ^12^C-benzoate instead of α-^13^C-benzoate was used. For the cultivation of *Escherichia coli* BL21(DE3), the medium was supplemented with 5 mM sodium ^12^C-acetate in addition to 5 mM benzoate. Cells from pure cultures were harvested during the early stationary phase as determined by photometric monitoring of the turbidity at 600 nm (OD_600_).

For co-cultivation of *E. coli* and *T*. *aromatica*, the medium was first inoculated with 10^5^ cells mL^−1^ of *E. coli* (resulting cell number) and incubated for 45 h until the OD_600_ stagnated at approximately 0.25. Then, 10^6^ cells mL^−1^ of *T. aromatica* (resulting cell number) were injected, and the incubation was continued. Samples of 1 mL were taken before and after adding *T. aromatica*, as well as after 93, 105, 129 and 205 h total incubation time.

For co-cultivation of *P*. *putida* and *T. aromatica*, the medium was initially inoculated with 10^6^ cells mL^−1^ of *T. aromatica* (resulting cell number) and incubated for 15 h before injecting 10^5^ cells mL^−1^ of *P. putida* (resulting cell number) into the medium and continuing cultivation. Cell samples with a volume of 1 mL were collected before and after adding *P. putida* as well as after 24, 27, 39 and 46 h. The incubation times, supporting the growth of both microorganisms, were determined by analyzing the growth curves of the individual organisms.

Cells were harvested via centrifugation at 16,000 x g for 10 min and washed once with 50 mM ammonium bicarbonate (AMBIC) buffer, pH 7.9.

### Method details

#### Preparation of α-^13^C-benzoate

α-^13^C-benzoic acid (CAS number 3880-99-7, chemical purity: > 98%), in which the carboxyl group carbon is isotopically labeled, was purchased from Eurisotop, Germany, and aerobically dissolved in an aqueous 254 mM sodium hydroxide solution to prepare a 254 mM sodium α-^13^C-benzoate stock solution that was used during this study.

#### Protein extraction and sample preparation

To extract proteins, cell pellets were resuspended in 250 μL of 50 mM AMBIC buffer and lysed by three freeze and thaw cycles, using liquid nitrogen and a thermal shaker operating at 40°C. Then, samples were sonicated for 30 s in a sonication bath before centrifuging at 16,000 x g for 10 min to remove cell debris and insoluble proteins. Protein concentrations in the supernatant were determined with the bicinchoninic acid (BCA) kit (Pierce, Thermo Scientific, Waltham, MA, USA) following the enhanced protocol. To facilitate the detection of labeled peptides, crude protein extracts from cells grown with α-^13^C-benzoate were mixed with crude protein extracts from unlabeled controls in a 1:1 ratio (if not stated otherwise) based on their protein content to a total of 5 μg protein in 30 μL of 50 mM AMBIC buffer, followed by sample preparation for GroEL-proteotyping mass spectrometry.^40^

For in-solution digestion, 40 ng of bovine serum albumin (BSA) was added to the sample as a quality control measure. Then, samples underwent sequential treatment with dithiothreitol (final concentration: 62.5 mM) and 2-iodoacetamide (final concentration: 128 mM) to reduce and alkylate cysteine residues. Proteins were digested overnight with 0.126 μg of trypsin per μg protein (Promega, Madison, WI, USA). Afterward, we added formic acid to a final concentration of 1.8% (v/v) to halt the digestion. Subsequently, we removed undigested and precipitated proteins by centrifugation at 16,000 x g for 10 min. The peptide-containing supernatant was desalted by Pierce C-18 tips (Thermo Scientific, Waltham, MA, USA) and dried using a vacuum centrifuge.

For in-gel digestion, we added 10 μL SDS reducing buffer to the crude extracts and heated the mixture for 10 min at 95°C. Then, we subjected the samples to SDS-PAGE^61^ at 110 V for 60–90 min and stained them with Coomassie G-250.^62^ Subsequently, we cut-out protein bands with a molecular weight of approximately 60 kDa, corresponding to the size of GroEL, and performed in-gel digestion following established protocols.^24^ For this purpose, we first destained gel slices with acetonitrile and reduced the proteins with 50 μL of 10 mM dithiothreitol before alkylating them with 50 μL of 100 mM 2-iodoacetamide. Then, we added 40 ng of reduced and alkylated BSA for quality control. Subsequently, proteins were digested with 0.1 μg of trypsin (Promega, Madison, WI, USA) at 37°C overnight. Afterward, we extracted peptides from the gel pieces using 50% (v/v) acetonitrile and 5% (v/v) formic acid before drying the peptides by vacuum centrifugation. The peptides were then resuspended in 20 μL of 0.1% (v/v) formic acid, desalted by Pierce C-18 tips (Thermo Scientific, Waltham, MA, USA), and dried again using a vacuum centrifuge.

#### Mass spectrometry

After trypsin digestion, desalted peptides were resuspended in 50 μL of 0.1% (v/v) formic acid and analyzed by nLC-MS/MS using an UltiMate 3000 RSLCnano high-performance nano-UPLC system (Thermo Scientific, Waltham, MA, USA) coupled to an Orbitrap Fusion Tribrid mass spectrometer (Thermo Scientific, Waltham, MA, USA) via a TriVersa NanoMate nano-electrospray ionization (nano-ESI) ion source (Advion, Ithaca, NY, USA) as described previously.^40^ In brief, peptides were separated at 35°C on a Acclaim PepMap 100 C_18_ analytical column (75 μm × 25 cm, 3 μm material, Thermo Scientific, Waltham, MA, USA) using a gradient from 3.2% to 72% (v/v) acetonitrile in water at 0.1% (v/v) formic acid with a flow rate of 0.3 μL min^−1^ for 60 min or 145 min, for in-gel and in-solution digested samples, respectively. Precursor ions were measured in the Orbitrap analyzer at a resolution of 120,000. Precursor ions with an intensity of at least 5 × 10^4^ were selected and fragmented via higher energy collisional dissociation (HDC) at 30% relative collision energy. Fragments were measured in the Orbitrap analyzer at a resolution of 30,000 or 60,000 for in-solution and in-gel digested samples, respectively. We enabled the dynamic exclusion of precursor ions of the same mass (± 10 ppm) for 30 s.

### Quantification and statistical analysis

Mass spectrometric raw files were converted into mzML format using msConvert (ProteoWizard).^59^ Subsequently, mzML files were analyzed by a customized MetaProSIP workflow^63^ using OpenMS^60^ on the Galaxy platform^57^ with our GroEL database containing 72,759 non-redundant sequences that were downloaded from the National Center for Biotechnology Information (NCBI) on September 7, 2021. In brief, peptides were identified by MS-GF+^58^ and their relative isotope abundance (RIA) and labeling ratios (LR) were determined by MetaProSIP^53^ using the following parameters: up to two missed trypsin cleavages were allowed, peptide lengths of 6–40 amino acids, precursor *m/z* deviation tolerance of ±5 ppm, weight merge window of ±1.5 (for ^2^H) or ±5.0 (for ^18^O and ^13^C), methionine oxidation as dynamic modification as well as carbamidomethylation of cysteine as static modification, false discovery rate below 0.01 (based on the SpecEValue calculated by MS-GF+). While the RIA describes the abundance of ^13^C isotopes in the labeled version of a given peptide, the LR describes the abundance of the labeled version of a peptide among the sum of the labeled and unlabeled version of this peptide.^24^ The report of unlabeled peptides was enabled. The peptide-centric output of MetaProSIP was used as input for the updated GroEL-proteotyping Python script (v2.0.0).^64^

The GroEL-proteotyping Python script v2.0.0 assigns identified unlabeled GroEL peptides to GroEL protein groups, filters them by a Top Rank Count threshold of ≥5 to minimize false-positive identifications and infers taxonomy as well as relative taxa abundances as previously described,^40^ but now also evaluates the number, intensity, labeling ratio (LR) and relative isotope abundance (RIA) of isotopically labeled peptides assigned to the identified taxa. More precisely, peptides are designated as isotopically labeled when MetaProSIP calculates an RIA beyond the natural RIA (RIA 1). Furthermore, peptides are considered as “shared” when they are present in multiple taxonomic groups. For each taxonomic group, here at genus or family level, the relative number of non-shared isotopically labeled peptides to non-shared labeled and non-labeled peptides is calculated as the relative count of labeled peptides (RA). Furthermore, the median LR and median RIA of isotopically labeled peptides assigned to a taxon are calculated from the MetaProSIP output. If MetaProSIP reported multiple RIAs for a peptide, we only used the highest RIA to calculate the median RIA and the median LR. Taxa were quantified by the sum of the MS1 precursor ion intensities (INT) calculated by MetaProSIP of labeled and unlabeled peptides within the taxonomic group. The precursor intensity of shared labeled or unlabeled peptides was distributed proportionately to the total number of labeled or unlabeled peptides assigned to each group, respectively. To evaluate our workflow, we used published metaproteome data from an *in vitro* model of the human distal gut. The accession number is listed in the key resources table.

Statistical details of experiments can be found in the results section and the figure legends. Significance was defined based on Student’s t test on the means of replicates at *p* < 0.05 (∗); *p* < 0.01 (∗∗); and *p* < 0.001 (∗∗∗), respectively.

## Supplemental information

Document S1. Tables S1 and S4

Table S2. Data and statistics on GroEL-based stable isotope probing of a co-cultivated biculture consisting of *Escherichia coli* BL21(DE3) and *Thauera aromatica* K172 at different time points used to generate Figure 1

Table S3. Data and statistics on GroEL-based stable isotope probing of a co-cultivated biculture consisting of *Thauera aromatica* K172 and *Pseudomonas putida* KT2440 at different time points used to generate Figure 2

Table S5. Detailed information on individual peptides identified by GroEL-based stable isotope probing of an *in vitro* model of the human gut cultivated with a fiber-rich or protein-rich medium in the presence of unlabeled H_2_O (S5A), ^2^H_2_O (S5B), or H_2_^18^O (S5C), related to Figure 3
