# Supplementary material for: Revealing taxonomy, activity, and substrate assimilation in mixed bacterial communities by GroEL-proteotyping-based stable isotope probing
Source: iScience. 2024 Oct 28;27(12):111249. doi: 10.1016/j.isci.2024.111249 (PMC11700628; doi:10.1016/j.isci.2024.111249)
Supplement: Document S1. Tables S1 and S4 [file mmc1.pdf]

iScience, Volume *27*

Supplemental information

Revealing taxonomy, activity, and substrate assimilation in mixed bacterial communities by GroEL-proteotyping-based stable isotope probing

Simon Klaes, Shobhit Madan, Darja Deobald, Myriel Cooper, and Lorenz Adrian

SUPPLEMENTARY TABLE S1. The number of identified peptides as well as relative isotope abundance (RIA) and labeling ratio (LR) of labeled peptides from protein extracts of organisms cultivated in DSMZ 586 medium with 5 mM α-^{13}C-benzoate or ^{12}C-benzoate mixed in a 50:50 ratio. For *Escherichia coli* BL21(DE3) cultivation, the medium was additionally supplemented with 42 mM ^{12}C-acetate. Peptides were identified using the whole proteome database of *Thauera aromatica* K172, *Pseudomonas putida* KT2440, or *E. coli* BL21(DE3) containing 3,335; 5,450; and 4,061 entries, respectively. Peptides were counted as labeled if at least one RIA value was detected in addition to the natural RIA. If MetaProSIP provided multiple RIA values in addition to the natural RIA for a peptide, we only used the highest RIA to calculate the median and LR. Values represent means of biological triplicates ± standard deviations. Related to Results.

Organism	Total identified peptides	Identified labeled peptides	Median RIA of labeled peptides [%]	Median LR of labeled peptides [%]
T. aromatica K172	1549.1 ± 356.5	743.7 ± 171.5	12.2 ± 0.0	50.9 ± 3.0
P. putida KT2440	1550.3 ± 419.2	1.0 ± 1.0	2.2 ± 1.9	33.3 ± 28.9
E. coli BL21(DE3)	1702.0 ± 389.5	1.7 ± 0.6	7.1 ± 2.8	46.3 ± 14.9

SUPPLEMENTARY TABLE S4. Data and statistics on GroEL-based stable isotope probing of an *in vitro* model of the human gut cultivated with a fiber-rich or protein-rich medium used to generate Figure 3.

	unlabeled controls						^{2}H labeling								^{18}O labeling							
	high fiber(3,4,5)			high protein(6,7,8)			high fiber(9,10,11)			high protein(12,13,14)			fiber vs protein		high fiber(15,16,17)			high protein(18,19,20)			fiber vs protein	
**Family**	Mean relative abundance (unlabeled)	Mean labeled subset	Mean RIA of labeled peptides	Mean relative abundance (unlabeled)	Mean labeled subset	Mean RIA of labeled peptides	Mean relative abundance (unlabeled)	Mean labeled subset	Mean RIA of labeled peptides	Mean relative abundance (unlabeled)	Mean labeled subset	Mean RIA of labeled peptides	p-value (labeled subset)	p-value (RIA)	Mean relative abundance (unlabeled)	Mean labeled subset	Mean RIA of labeled peptides	Mean relative abundance (unlabeled)	Mean labeled subset	Mean RIA of labeled peptides	p-value (labeled subset)	p-value (RIA)
Akkermansiaceae	0.175	0.000	x	0.214	0.000	x	0.214	0.000	x	0.194	0.039	6.317	0.009	x	0.330	0.242	22.458	0.294	0.215	20.692	0.130	0.339
Bacteroidaceae	0.168	0.000	x	0.153	0.000	x	0.253	0.041	4.017	0.134	0.079	3.900	0.015	0.890	0.215	0.121	19.492	0.112	0.080	19.283	0.135	0.896
Enterobacteriaceae	0.003	0.000	x	0.014	0.000	x	0.000	0.000	x	0.000	0.000	x	x	x	0.000	0.000	x	0.000	0.000	x	x	x
Eubacteriaceae	0.015	0.000	x	0.047	0.000	x	0.045	0.030	4.067	0.053	0.018	4.733	0.084	0.029	0.026	0.019	15.825	0.072	0.062	16.550	0.027	0.410
Erysipelotrichaceae	0.020	0.000	x	0.008	0.000	x	0.065	0.015	2.783	0.000	0.000	x	x	x	0.032	0.016	13.433	0.000	0.000	x	x	x
Clostridiaceae	0.000	0.000	x	0.011	0.000	x	0.000	0.000	x	0.000	0.000	x	x	x	0.000	0.000	x	0.000	0.000	x	x	x
Christensenellaceae	0.000	0.000	x	0.002	0.000	x	0.000	0.000	x	0.000	0.000	x	x	x	0.000	0.000	x	0.000	0.000	x	x	x
Lachnospiraceae	0.217	0.000	x	0.275	0.001	10.000	0.300	0.032	4.917	0.382	0.136	3.842	0.001	0.019	0.344	0.172	18.083	0.315	0.163	15.600	0.835	0.148
Rikenellaceae	0.344	0.000	x	0.108	0.001	8.000	0.051	0.011	2.850	0.088	0.028	2.883	0.232	0.942	0.000	0.000	x	0.085	0.052	17.500	x	x
Ruminococcaceae	0.036	0.001	2.900	0.015	0.000	x	0.055	0.019	5.692	0.000	0.000	x	x	x	0.061	0.047	16.775	0.000	0.000	x	x	x
Sutterellaceae	0.001	0.000	x	0.001	0.000	x	0.000	0.000	x	0.000	0.000	x	x	x	0.000	0.000	x	0.000	0.000	x	x	x
Synergistaceae	0.000	0.000	x	0.050	0.000	x	0.000	0.000	x	0.052	0.000	x	x	x	0.000	0.000	x	0.064	0.005	18.150	x	x
Oscillospiraceae	0.021	0.000	x	0.102	0.000	x	0.017	0.000	x	0.096	0.032	3.933	x	x	0.020	0.000	x	0.059	0.035	16.250	x	x
